# Supplementary material for: Chemical signal activation of an organocatalyst enables control over soft material formation
Source: Nat Commun. 2017 Oct 12;8:879. doi: 10.1038/s41467-017-00998-3 (PMC5638897; doi:10.1038/s41467-017-00998-3)
Supplement: Supplementary file 1 — Supplementary Information [file 41467_2017_998_MOESM1_ESM.pdf]

Supplementary Figures

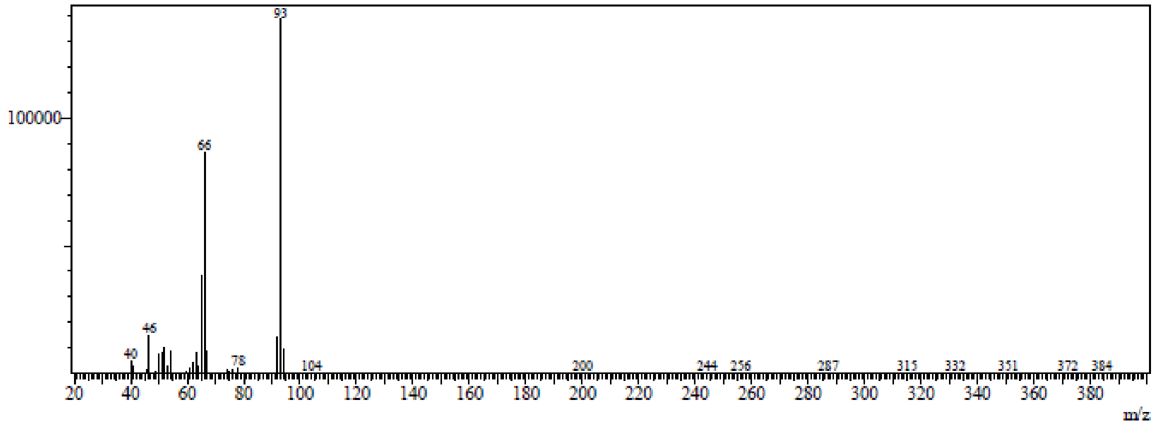

Supplementary Figure 1: GC/MS spectrum taken after the reaction of pro-aniline **1** with 18 equivalents of H_2O_2. Conditions: 5 mg, 18 µmol pro-aniline **1** in 0.25 mL ethyl acetate, H_2O_2 (18 equivalents, 25.4 µL in 0.25 mL deionized water), stirred for 1 h at room temperature. The reaction mixture was quenched with a saturated sodium thiosulfate solution and the organic layer was used for analysis in GC/MS. Only aniline **2** was detected in the reaction mixture, MS (GC/MS) m/z: 93 $[M]^{+\bullet}$, 66 $[C_5H_6]^{+\bullet}$ (expected m/z = 93.06, 66.05), retention time: 11 min.

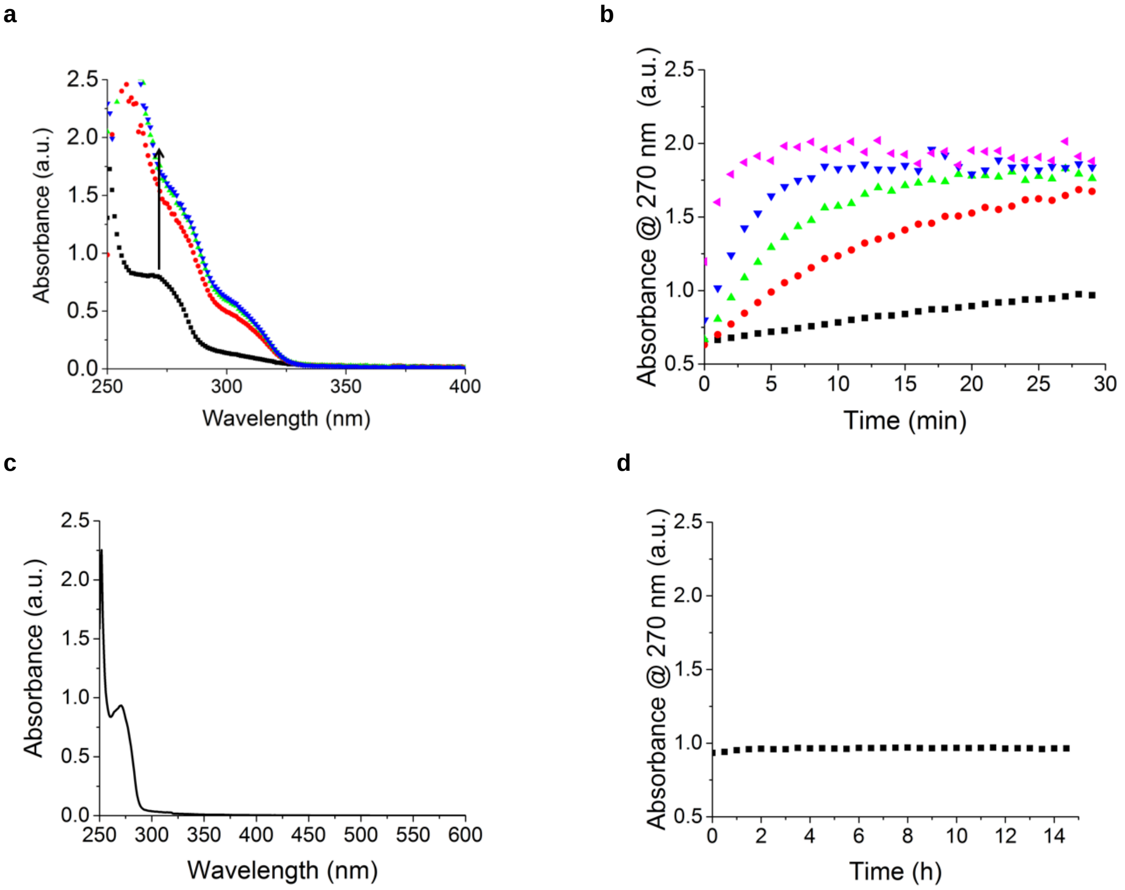

Supplementary Figure 2: The self-immolative reaction of pro-aniline **1** (0.5 mM) with H_2O_2 (9 mM) in 20% DMF in phosphate buffer (100 mM, pH 7.4) followed in UV/vis spectroscopy. (**a**) UV/vis absorption spectra of pro-aniline **1** after addition of H_2O_2, spectrum at t = 0 min (black), spectrum at t = 5 min (red), spectrum at t = 10 min (green), spectrum at t = 15 min (blue). (**b**) The increase of absorption at 270 nm after addition of H_2O_2 to a solution of **1**, 1 equivalent of H_2O_2 (black), 5 equivalents (red), 10 equivalents (green), 18 equivalents (blue), 50 equivalents (pink). (**c**) The UV/vis absorption spectrum of pro-aniline **1**. (**d**) Without H_2O_2 the absorption spectrum of pro-aniline **1** does not change in 15 h.

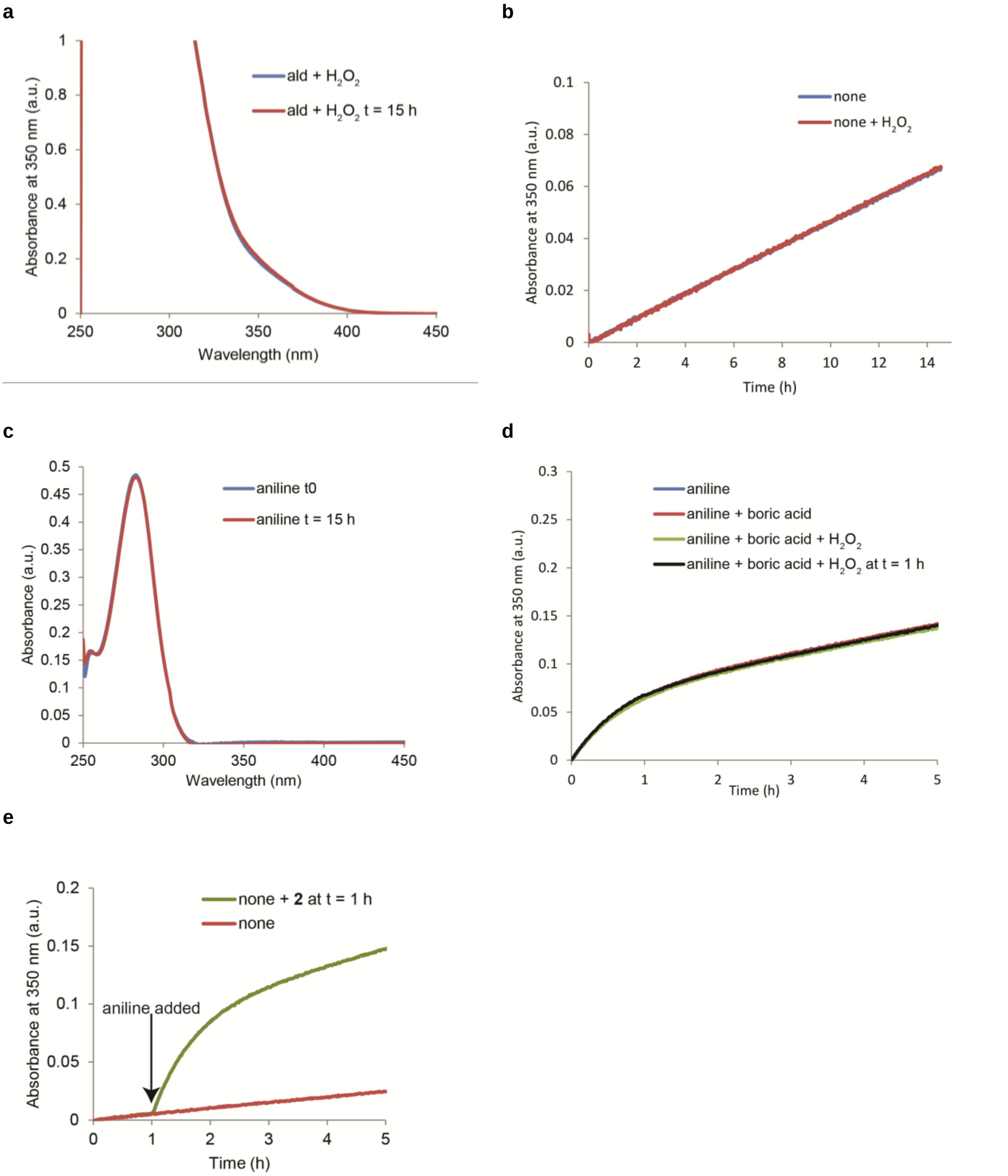

Supplementary Figure 3: (**a**) UV/vis absorbance spectrum measured for aldehyde **4** (0.5 mM) with H_2O_2 at t = 0 h and at t = 15 h, the compound remains stable. (**b**) Absorbance at 350 nm of hydrazone **5** formation followed in UV/vis spectroscopy (conditions: hydrazide **3** (0.1 mM), aldehyde **4** (0.5 mM), 20% DMF in 100 mM phosphate buffer pH 7.4) without catalyst (blue line), with H_2O_2 (2.5 mM, red line). H_2O_2 has no influence on the reaction rate. (**c**) UV/vis absorbance spectrum measured for aniline **2** (0.5 mM) at t = 0 h and at t = 15 h, the compound remains stable. (**d**) Absorbance at 350 nm of hydrazone **5** formation followed in UV/vis spectroscopy (conditions: hydrazide **3** (0.1 mM), aldehyde **4** (0.5 mM), 20% DMF in 100 mM phosphate buffer pH 7.4) with aniline **2** (0.5 mM, blue line), with aniline **2** + boric acid (0.5 mM, red line), with aniline **2** + boric acid + H_2O_2 (2.5 mM, green line), with aniline **2** + boric acid + H_2O_2 at t = 1 h (2.5 mM, black line. Boric acid has no influence on the catalytic activity of aniline **2**, with and without H_2O_2. (**e**) Absorbance at 350 nm of hydrazone **5** formation followed in UV/vis spectroscopy (conditions: hydrazide **3** (0.1 mM), aldehyde **4** (0.5 mM), 20% DMF in 100 mM phosphate buffer pH 7.4) without catalyst (red line), aniline **2** was added after 1 hour (green line, 2.5 mM. For more blank experiments, see Supplementary Table 1.

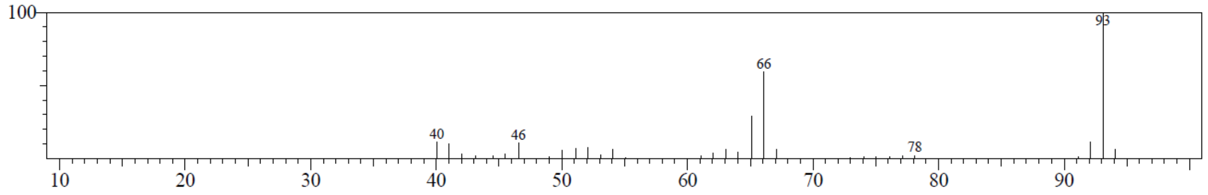

Supplementary Figure 4: GC/MS spectrum taken after 65 h of reaction time of hydrazide **3** with aldehyde **4** in the presence of pro-aniline **1** and H_2O_2. Conditions: hydrazide **3** (20 mM), aldehyde **4** (20 mM), pro-aniline **1** (10 mM), H_2O_2 (11 mM) in 20% DMF in 100 mM phosphate buffer pH 7.4. The reaction mixture was extracted after 65 h of reaction time with dichloromethane. The organic layer was evaporated and re-dissolved in ethyl acetate for GC/MS analysis. Aniline **2** was detected in the extract of the reaction mixture, MS (GC/MS) m/z: 93 $[M]^{+\bullet}$, 66 $[C_5H_6]^{+\bullet}$ (expected m/z = 93.06, 66.05), retention time: 11 min.

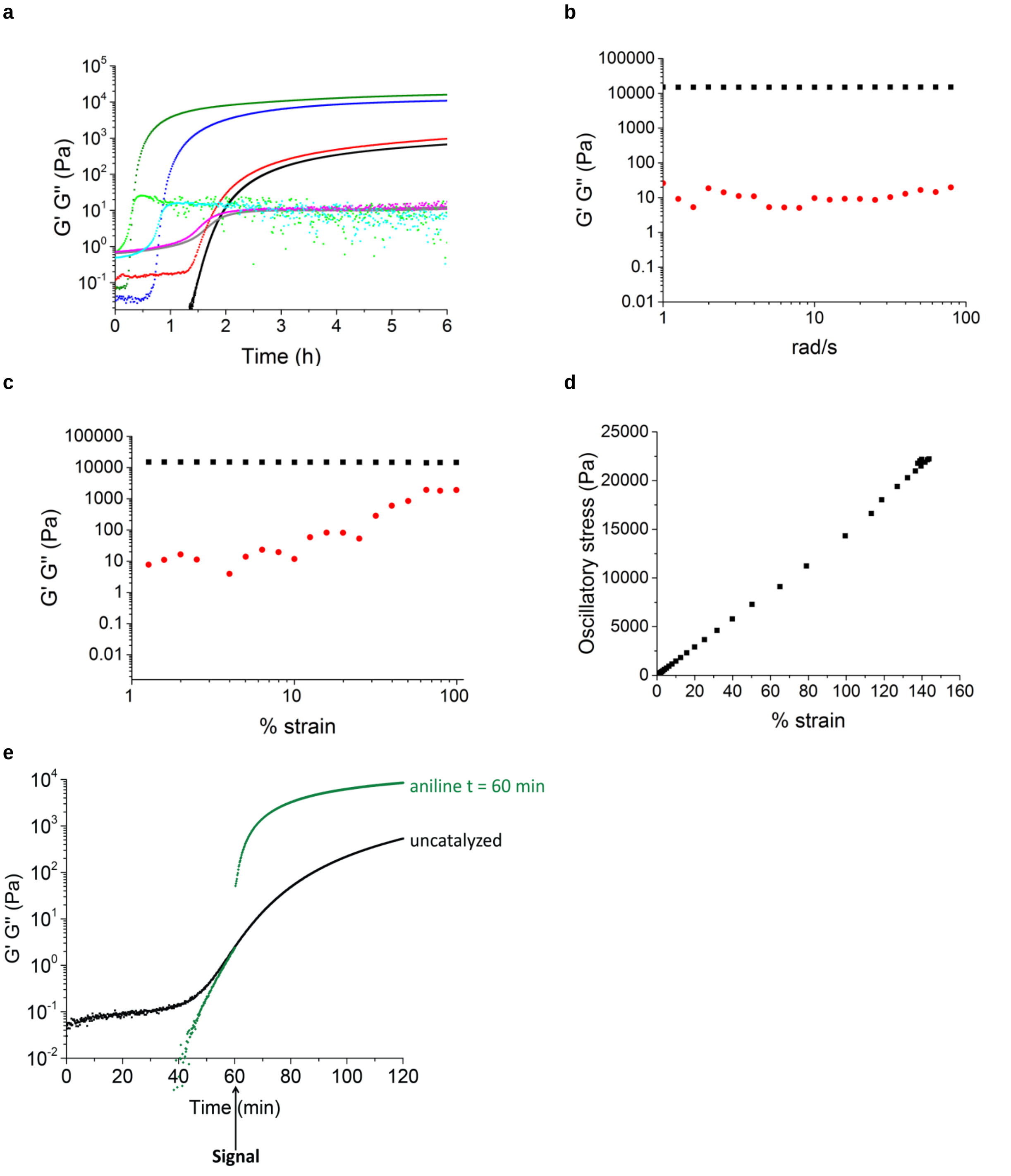

Supplementary Figure 5: Rheological data of the polymer gel. Total volume of the gels is 0.6 mL. Composition of the gels: 140 mg/mL polymer aldehyde **6**, 10 mM aniline **2** or 10 mM pro-aniline **1**, 10 mM hydrazide **7**, 20% DMF in 100 mM phosphate buffer pH 6.0. (**a**) Time sweep, *G'* and *G"* measured during the formation of the gel, uncatalyzed *G'* (black), uncatalyzed *G"* (grey), aniline **2** *G'* (green), aniline **2** *G"* (light green), pro-aniline **1** *G'* (red), pro-aniline **1** *G"* (pink), pro-aniline **1** + H_2O_2 (blue), pro-aniline **1** + H_2O_2 (light blue). (**b**) Frequency sweep of polymer gel formed with aniline **2** (10 mM) *G'* (black), *G"* (red). (**c**) Strain sweep of polymer gel formed with aniline **2** (10 mM), *G'* (black), *G"* (red). (**d**) Stress/strain curve of polymer gel formed with aniline **2** (10 mM) *G'* (black), *G"* (red). (**e**) Time sweep, *G'* and *G"* measured during the formation of the gel (total volume 1 mL), aniline **2** is added after 60 minutes, uncatalyzed (black), catalysed and aniline **2** (50 µL, 200 mM) added at *t* = 60 min (green).

a b

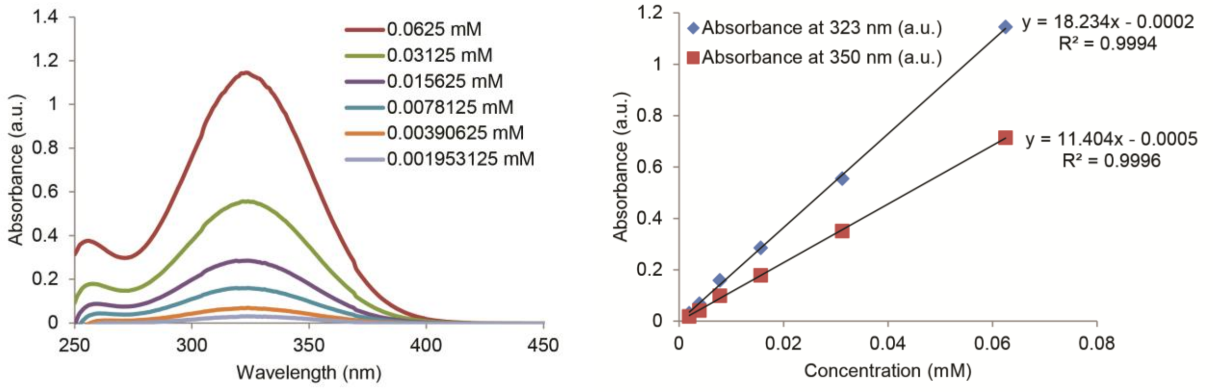

Supplementary Figure 6: (**a-b**) Calibration line of hydrazone **5** measured in 20% DMF in phosphate buffer (100 mM, pH 7.4). The extinction coefficient of hydrazone **5** under these conditions at the absorbance maximum of 323 nm is 2.1 ± 0.13 · 10^4 M^{-1} s^{-1}. The extinction coefficient at 350 nm is 1.3 ± 0.087 · 10^4 M^{-1} s^{-1}. The errors are the standard error of mean (the standard deviation divided by the square root of the number of measurements).

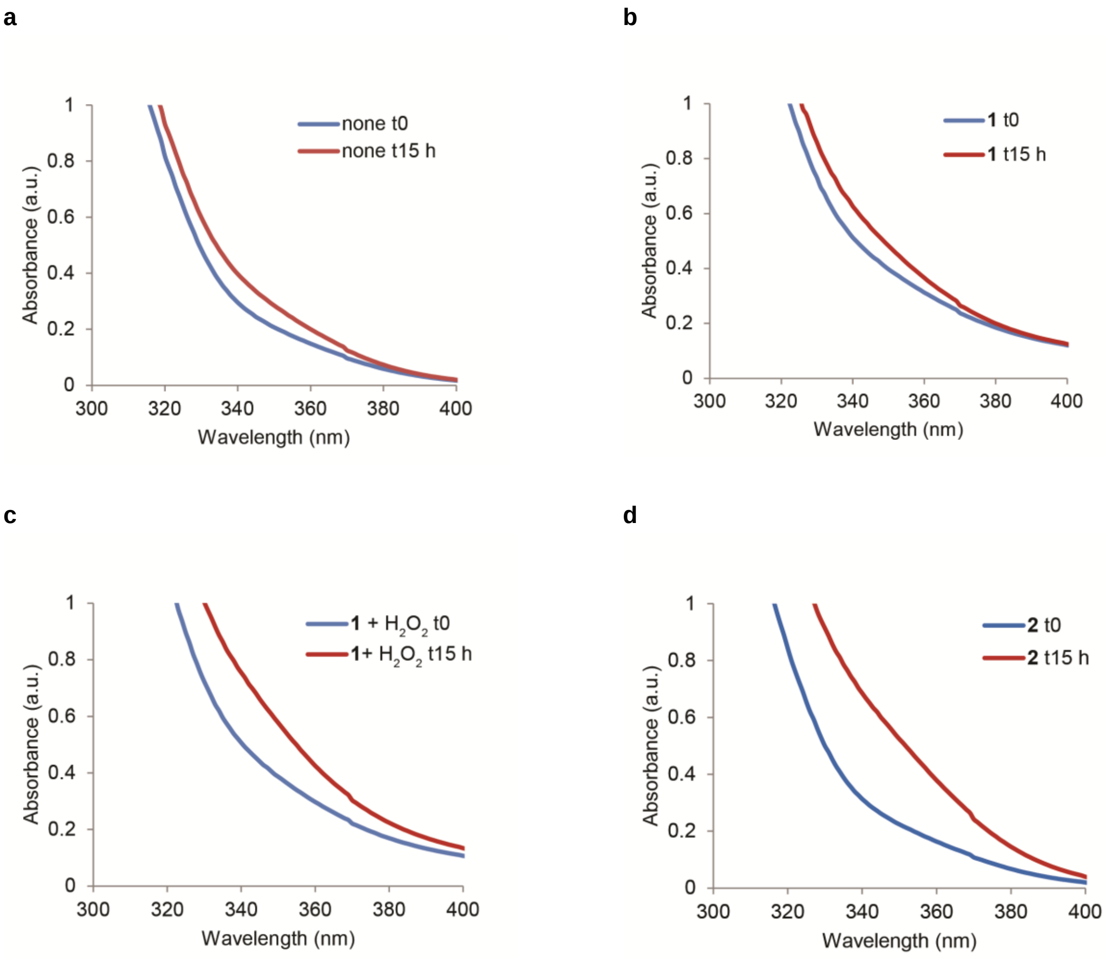

Supplementary Figure 7: UV/vis spectra of the reaction mixture of hydrazone formation at t = 0 h (blue lines) and at t = 15 h (red lines). (**a**) Uncatalyzed reaction, (**b**) reaction in presence of pro-aniline **1**, (**c**) reaction in presence of pro-aniline **1** and H_2O_2, (**d**) reaction in the presence of aniline **2**. Reaction conditions: 0.1 mM hydrazide **3**, 0.5 mM aldehyde **4** in 20% DMF in phosphate buffer (100 mM, pH 7.4) without catalyst, in the presence of either unactivated pro-aniline **1** (0.5 mM), or activated pro-aniline **1** (0.5 mM) with 5 equivalents of H_2O_2 (2.5 mM) or aniline **2** (0.5 mM).

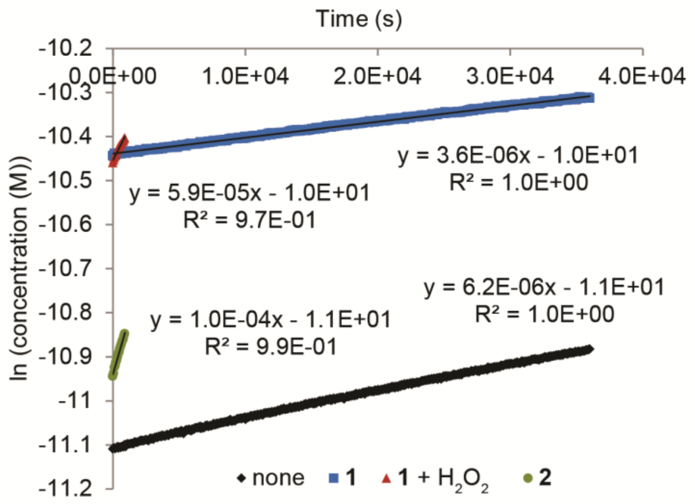

Supplementary Figure 8: Plots of ln (concentration (M)) versus time (s) result in linear graphs, the slope represents the pseudo-first-order rate constant with unit s^{-1}, without catalyst (black), with pro-aniline **1** (blue), with pro-aniline **1** + H_2O_2 (red) and with aniline **2** (green). Concentrations were obtained using the extinction coefficient of the product at 350 nm ($1.3 \pm 0.087 \cdot 10^4$ M^{-1} s^{-1}). The errors are the standard error of mean (the standard deviation divided by the square root of the number of measurements). Reaction conditions: 0.1 mM hydrazide **3**, 0.5 mM aldehyde **4** in 20% DMF in phosphate buffer (100 mM, pH 7.4) without catalyst, in the presence of either unactivated pro-aniline **1** (0.5 mM), or activated pro-aniline **1** (0.5 mM) with 5 equivalents of H_2O_2 (2.5 mM) or aniline **2** (0.5 mM).

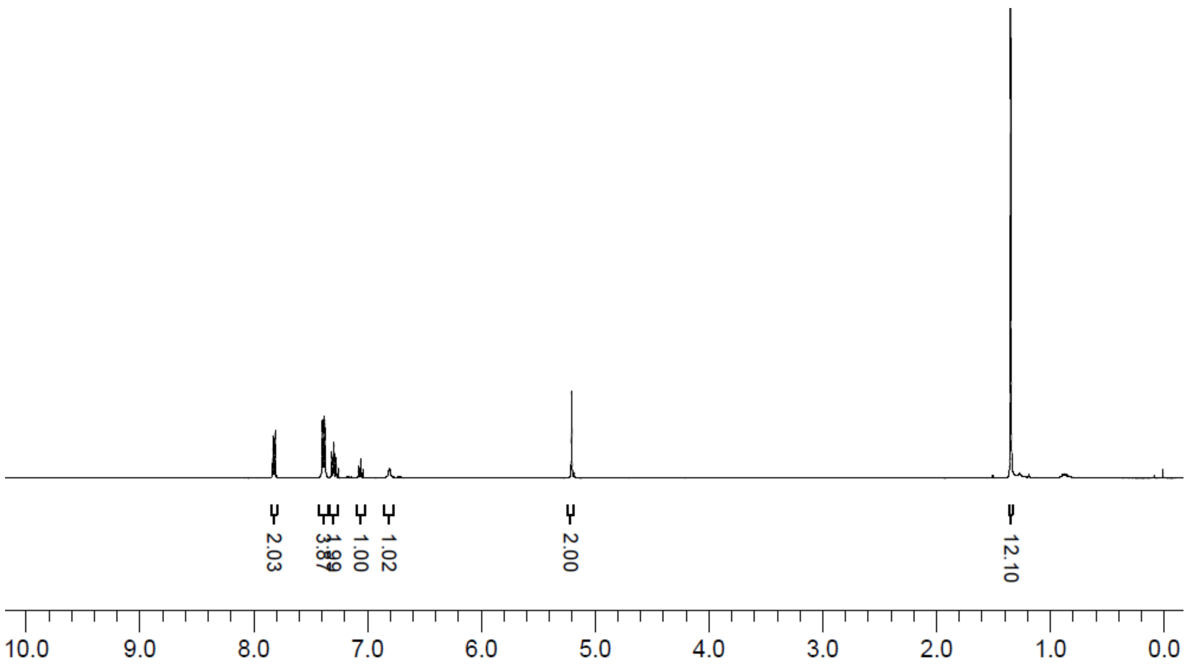

Supplementary Figure 9: ^{1}H NMR spectrum of pro-aniline boronate ester **S1** in $CDCl_3$.

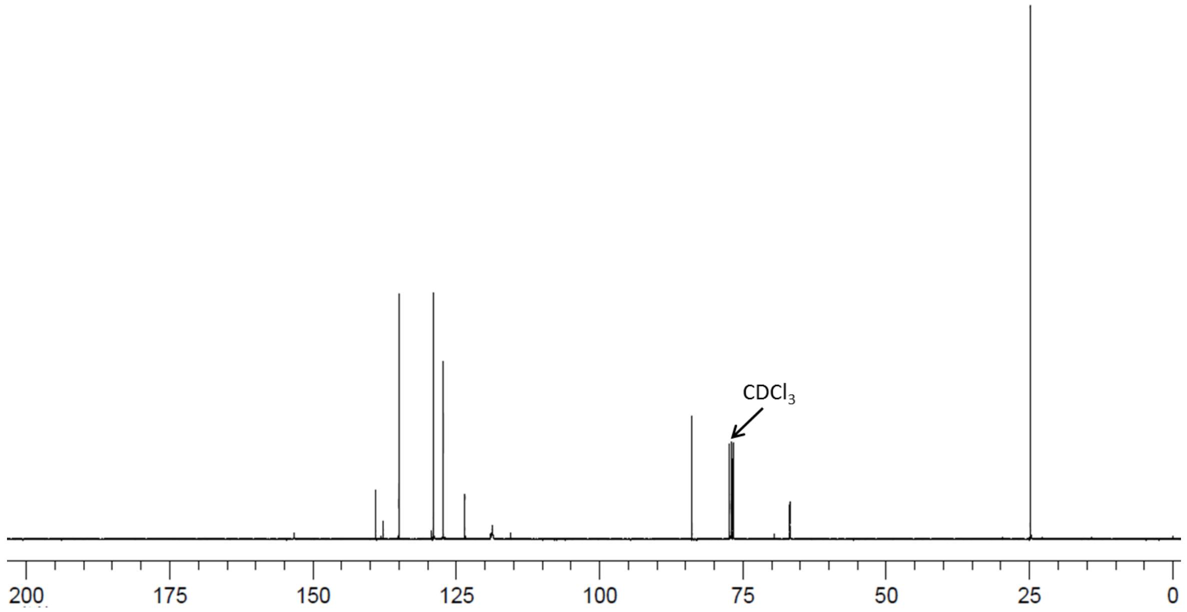

Supplementary Figure 10: ^{13}C NMR spectrum of pro-aniline boronate ester **S1** in $CDCl_3$.

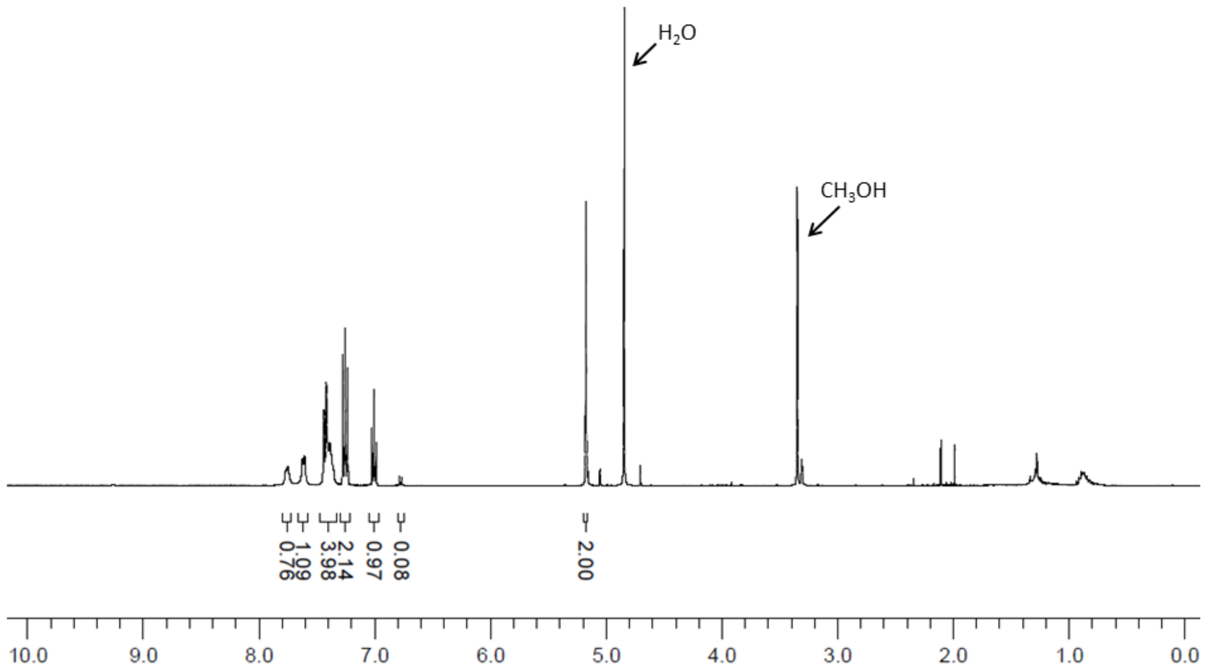

Supplementary Figure 11: ^{1}H NMR spectrum of pro-aniline **1** in CD_3OD.

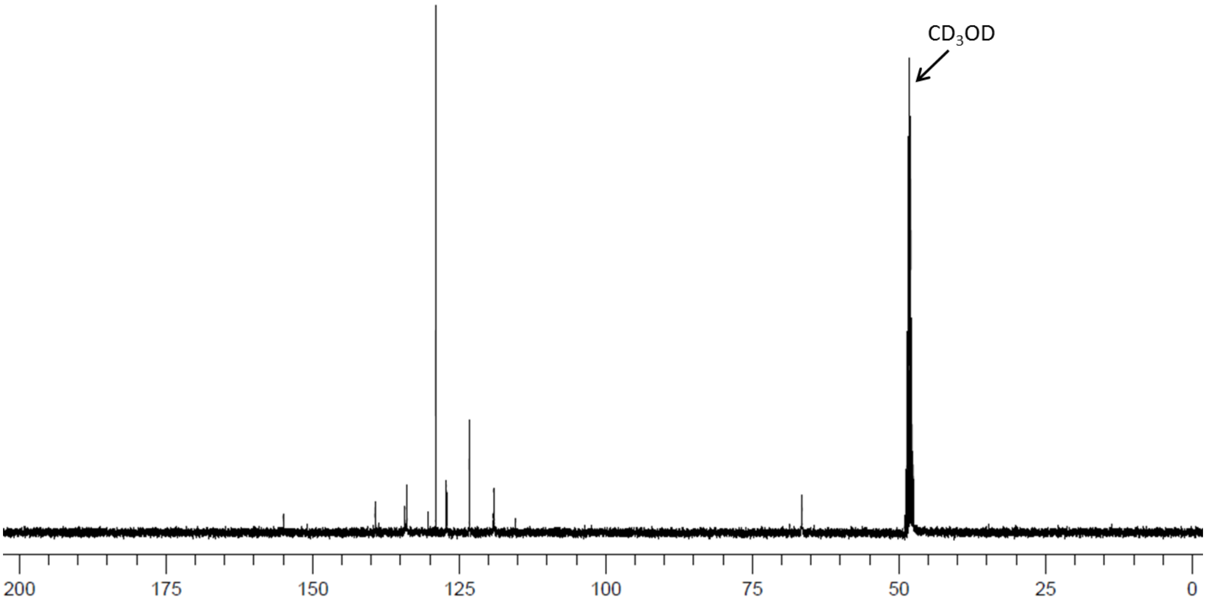

Supplementary Figure 12: ^{13}C NMR spectrum of pro-aniline **1** in CD_3OD.

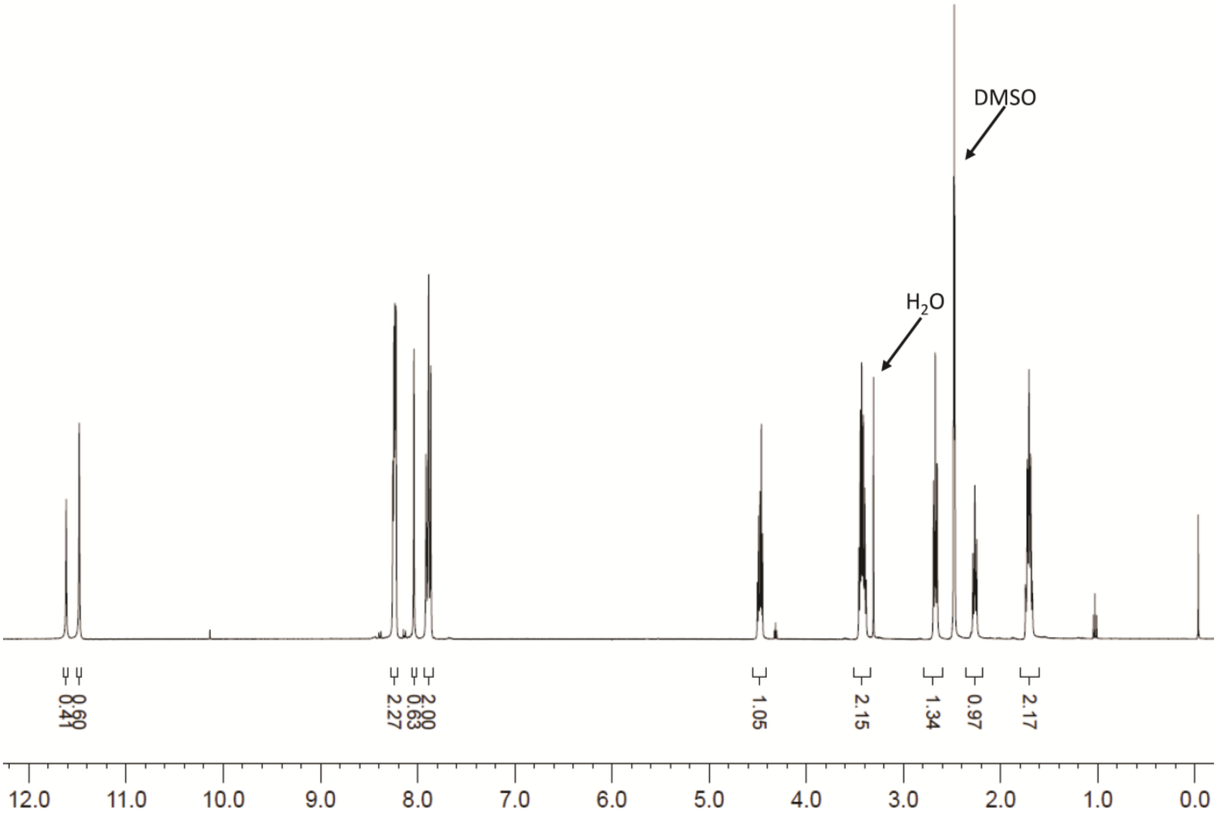

Supplementary Figure 13: ^{1}H NMR spectrum of hydrazone **5** in DMSO-d6.

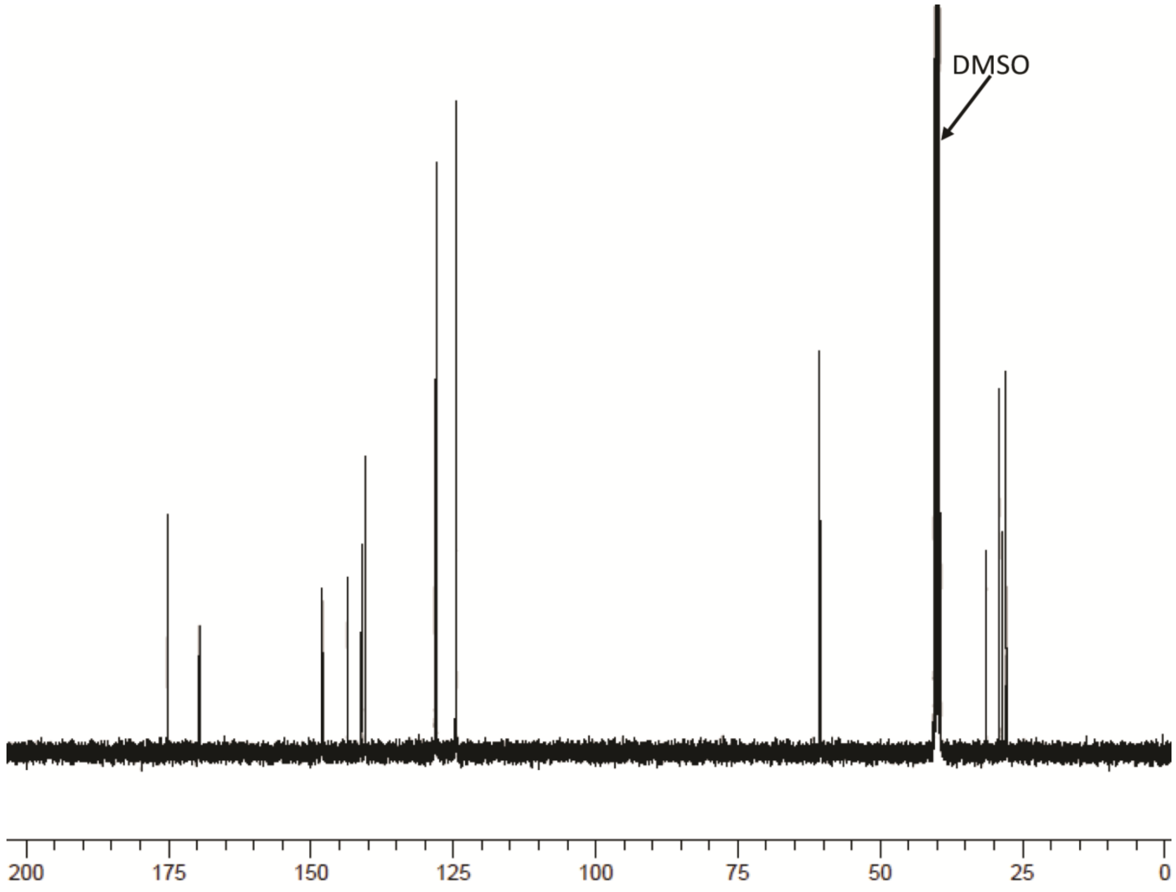

Supplementary Figure 14: ^{13}C NMR spectrum of hydrazone **5** in DMSO-d6.

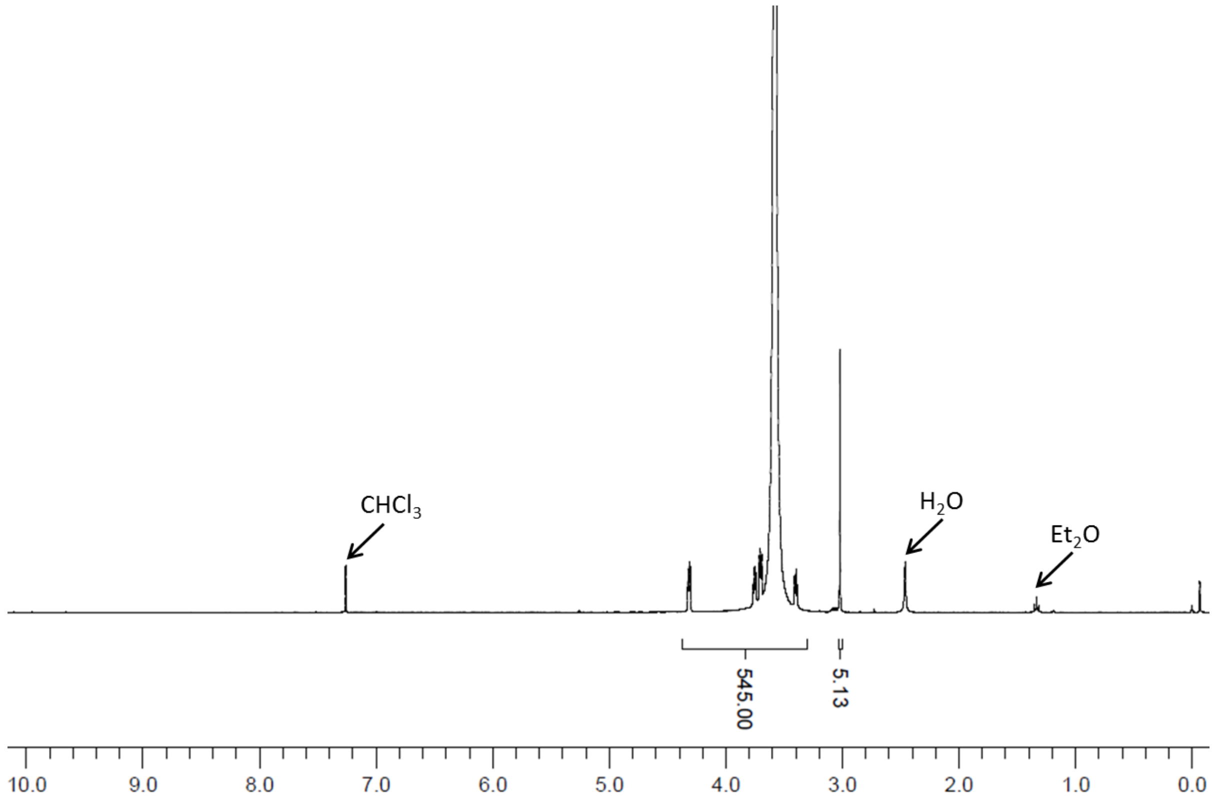

Supplementary Figure 15: ^{1}H NMR spectrum of PEG-mesylate **S2** in $CDCl_3$.

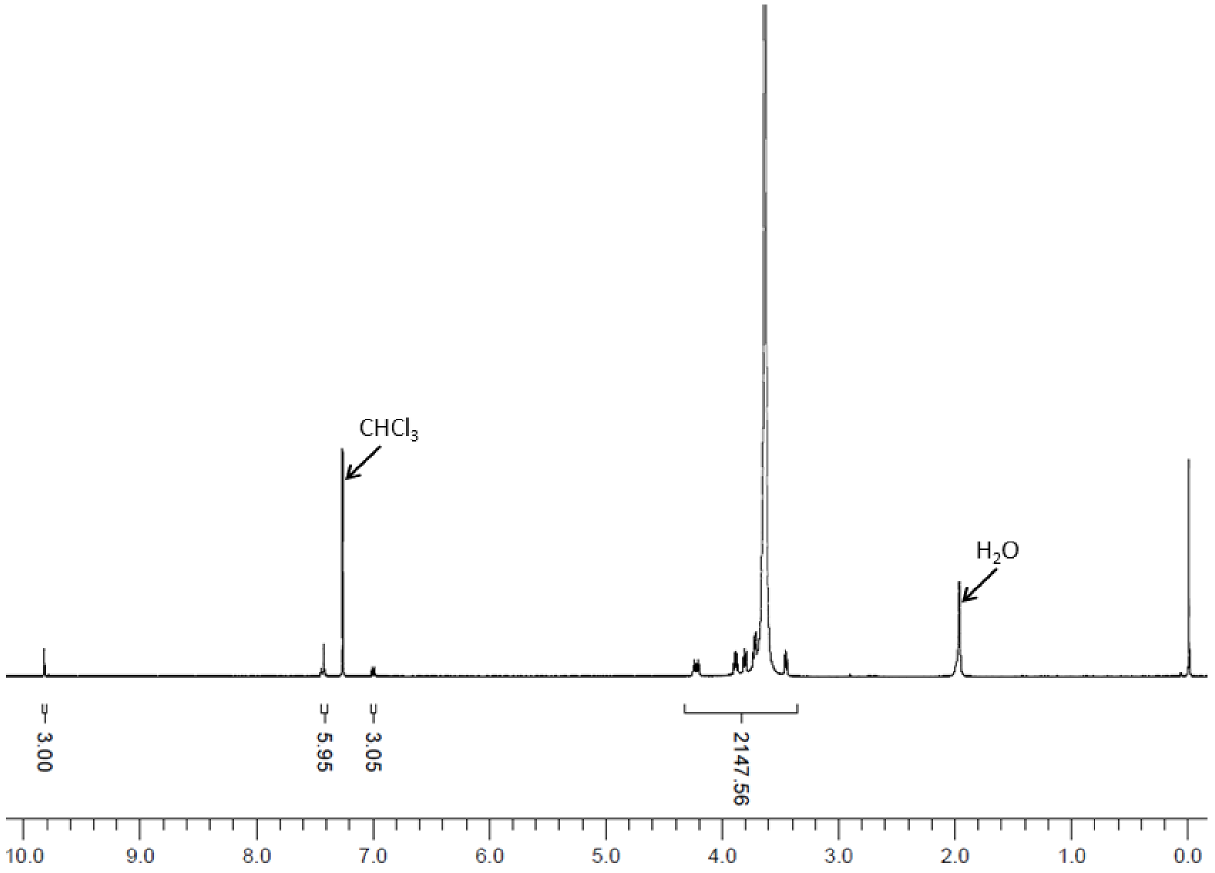

Supplementary Figure 16: ^{1}H NMR spectrum of PEG-aldehyde **6** in $CDCl_3$.

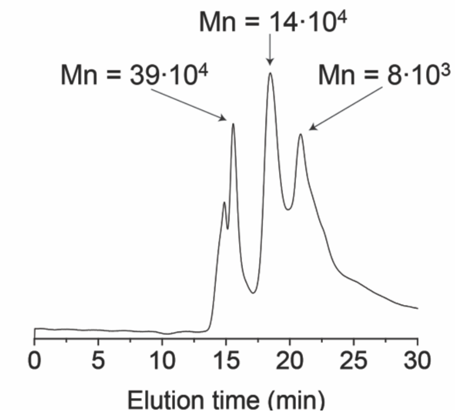

Supplementary Figure 17: Gel permeation chromatogram of PEG-aldehyde **6** (10 mg/mL in water).

Supplementary Tables

Supplementary Table 1: Overview of control experiments performed for the catalytic activity of hydrazone **5** formation. In all cases, the absorbance at 350 nm was followed during the reaction and spectra between 250 – 450 nm were taken at the start and at the end of the reaction.

#	Hydrazide **3**	Aldehyde **4**	Pro-aniline **1**	H_2O_2	Aniline **2**	Boric acid	Results
1	x						no change in absorbance, stable for 15 h
2	x			x			no reaction 15 h
3	x		x				no reaction 15 h
4	x		x	x			no reaction 15 h
5	x				x		no reaction 15 h
6	x	x		x			same as uncatalyzed reaction
7	x	x		x	x		same as reaction with aniline **2**
8	x	x	x		x		same as reaction with aniline **2**
9	x	x	x	x	x		proportional increase in reaction rate
10		x					no change in absorbance, stable 15 h
11		x		x			no reaction 15 h
12		x	x				no reaction 15 h
13		x	x	x			no reaction 15 h
14		x			x		iminium formation negligible at 350 nm
15				x	x		stable for 15 h
16			x				stable for 15 h
17			x	x			no change in absorbance at 350 nm
18	x	x			x	x	same as reaction with aniline **2**
19	x	x		x	x	x	same as reaction with aniline **2**

Supplementary Table 2: Pseudo-first-order reaction rates for hydrazone formation were determined by following the absorbance of hydrazone **5** in UV/vis spectroscopy. The rate constants were determined by plotting the natural logarithm of the concentration (M) over time (s), in which the obtained slope is equal to the first-order rate constant of hydrazone **5** formation.

Catalyst system	Slope·10^6	k_1 (10^{-6} s^{-1})	k_{rel}
none	6.1 ± 0.1	6.1 ± 0.1	1.0
H_2O_2	6.0 ± 0.3	6.0 ± 0.3	1.0
**1**	5.6 ± 0.7	5.6 ± 0.7	0.9
**1** + H_2O_2	60 ± 8	60 ± 8	10
**2**	113 ± 5	113 ± 5	19

Supplementary Methods

Materials

All compounds and solvents were used without further purification. The technical solvents were purchased from VWR and the reagent grade solvents were purchased from Sigma Aldrich. 4-(Hydroxymethyl)benzeneboronic acid pinacol ester and 4-hydroxybutyric acid hydrazide were purchased from Alfa Aesar. Triphosgene, sodium periodate, PEG-6000, trimethylamine, palladium on activated charcoal, hydrazine hydrate (64%), trimesic acid, diethylene glycol monomethyl ether and 3,4-dihydroxybenzaldehyde were purchased from Sigma Aldrich. Aniline **2**, 4-nitrobenzaldehyde, acetic acid, methanesulfonyl chloride, *p*-toluenesulfonic acid monohydrate and *p*-toluenesulfonyl chloride were purchased from Acros. 3,4-Dihydroxybenzaldehyde was purchased from AK Scientific. Special safety precautions should be taken for the reaction with triphosgene as this compound can release highly toxic phosgene gas. Triphosgene should be handled in a closed fume hood at all times, while wearing protective gloves and glasses. We also offer an alternative synthetic route to pro-aniline **1** that avoids the use of triphosgene[1]. Hydrogen gas is highly flammable; DMF, aniline **2**, hydrazine hydrate and diethylene glycol monomethyl ether are suspected carcinogens; hydrogen peroxide is corrosive: these compounds should be handled with care.

Synthesis

Synthesis of pro-aniline 1

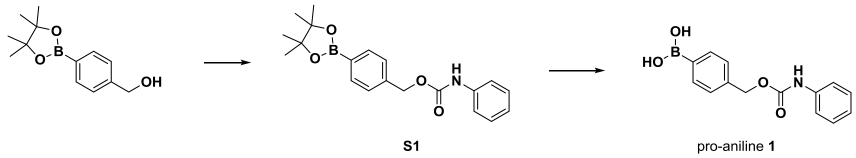

The 4-(4,4,5,5-tetramethyl-1,3,2-dioxaborolan-2-yl)benzyl phenylcarbamate (boronate ester derivative of pro-aniline **S1**) was synthesized using procedures described in the literature.[2, 3] The 4-(hydroxymethyl)phenylboronic acid pinacol ester was functionalized with triphosgene to form the chloroformate derivative. The boronate ester chloroformate was coupled to aniline **2** in the presence of a base ($NaHCO_3$). The boronic acid derivative pro-aniline **1** was obtained via elimination of pinacol using sodium periodate. Pro-aniline **1** can also be synthesized via the Curtius rearrangement of 4-formyl benzoic acid with diphenylphosphoryl azide and reaction with 4-(hydroxymethyl)phenylboronic acid pinacol ester and subsequent removal of the pinacol group.[1]

4-(4,4,5,5-Tetramethyl-1,3,2-dioxaborolan-2-yl)benzyl phenylcarbamate (S1)

First, K_2CO_3 (4.35 g, 31.5 mmol) was flame dried in a round-bottom flask. It was cooled in an ice bath and triphosgene (2.07 g, 7.0 mmol) in toluene was added. After stirring for 0.5 h at 0 °C, 4-(hydroxymethyl)phenylboronic acid pinacol ester (0.82 g, 3.5 mmol) in toluene was added. The mixture was allowed to reach ambient temperature and stirred overnight. After completion of the reaction, the reaction mixture was diluted with $CHCl_3$ and filtered through celite. The filtrate was washed with water, brine and dried over $MgSO_4$. Concentration under reduced pressure provided the chloroformate derivative which was used without further purification. To a stirred solution of aniline **2** (0.2 mL, 2.2 mmol) in THF at 0 °C $NaHCO_3$ was added (0.23 g, 2.7 mmol), followed by addition of the chloroformate derivative. After 1 h, the reaction mixture was allowed to reach ambient temperature, followed by addition of water to quench the reaction. The mixture was extracted with ethyl acetate (3x). The combined organic layers were dried over $MgSO_4$ and concentrated under reduced pressure to provide the desired compound (0.71 g, 90%). **^{1}H NMR** (Supplementary Figure 9) (400 MHz, $CDCl_3$): δ = 7.83 (d, *J* = 7.9 Hz, 2H, Ar-*H*), 7.40 (d, *J* = 7.9 Hz, 4H, Ar-*H*), 7.30 (t, *J* = 7.5 Hz, 2H, Ar-*H*), 7.06 (t, *J* = 7.4 Hz, 1H, Ar-*H*), 6.81 (s, 1H, -N*H*-), 5.21 (s, 2H, -CH_2-), 1.35 (s, 12H, CH_3). **^{13}C NMR** (Supplementary Figure 10) (100 MHz, $CDCl_3$): δ = 153.3 (-*C*OO-), 139.1 (C_{Ar}), 137.7 (C_{Ar}), 135.0 (C_{Ar}), 129.0 (C_{Ar}), 127.3 (C_{Ar}), 123.5 (C_{Ar}), 118.7 (C_{Ar}), 83.9 (-*C*Me_2-), 66.8 (-*C*H_2-), 24.8 (-*C*H_3). **Mp**: 113 – 115 °C. **MS** (GC/MS) m/z: 353 [M] (expected m/z = 353.2).

(4-(((Phenylcarbamoyl)oxy)methyl)phenyl)boronic acid (pro-aniline 1)

4-(4,4,5,5-tetramethyl-1,3,2-dioxaborolan-2-yl)benzyl phenylcarbamate **S1** (0.71 g, 2.0 mmol) was dissolved in acetone. $NaIO_4$ (3.42 g, 16.0 mmol) and ammonium acetate (1.23 g, 16.0 mmol) in water were added and the mixture was stirred overnight at room temperature. After completion of the reaction, the reaction mixture was acidified with 1M aqueous HCl and concentrated under reduced pressure. The crude substance was diluted with ethyl acetate and the mixture was washed with water, brine and dried over $MgSO_4$. Concentration under reduced pressure provided the desired compound as a light brown powder (0.45 g, 71%). **^{1}H NMR** (Supplementary Figure 11) (400 MHz, CD_3OD): δ = 7.75 (d, *J* = 7.3 Hz, 1H, Ar-*H*), 7.61 (d, *J* = 7.6 Hz, 1H, Ar-*H*), 7.42 – 7.34 (m, 4H, Ar-*H*), 7.26 – 7.21 (m, 2H, Ar-*H*), 7.01 – 6.97 (m, 1H, Ar-*H*), 5.17 (s, 2H, -CH_2-). **^{13}C NMR** (Supplementary Figure 12) (100 MHz, CD_3OD): δ = 155.9 (-*C*OO-), 140.3 (C_{Ar}), 135.2 (C_{Ar}), 134.9 (C_{Ar}), 131.2 (C_{Ar}), 129.9 (C_{Ar}),128.1 (C_{Ar}), 128.0 (C_{Ar}), 124.2 (C_{Ar}), 120.0 (C_{Ar}), 67.5 (-*C*H_2-). **Mp**: 189 – 190 °C. **MS** (ESI Neg.) m/z: 270.0 [$(M-H)^-$] (expected m/z = 271.1).

Synthesis 4-hydroxy-N'-(4-nitrobenzylidene)butanehydrazide hydrazone 5

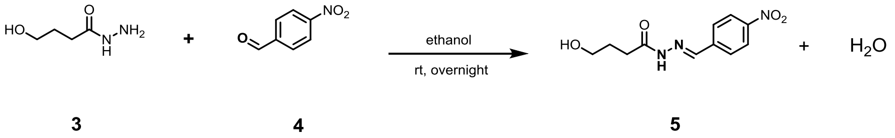

4-hydroxy-N'-(4-nitrobenzylidene)butanehydrazide was synthesized using a procedure reported in the literature.[4] 4-nitrobenzaldehyde **4** (129 mg, 0.855 mmol) and 4-hydroxybutyric acid hydrazide **3** (100 mg, 0.846 mmol) were dissolved in absolute ethanol (3 mL). A drop of acetic acid was added after which a yellow precipitate formed immediately and the reaction mixture was stirred at room temperature overnight. The precipitate was collected by filtration, washed with ethanol and dried in a vacuum oven at 40 °C to yield a fluffy yellow powder. Yield: 0.19 g, 0.75 mmol, 89%: a mixture of the *cis*- and *trans*-isomers. **^{1}H NMR** (Supplementary Figure 13) (400 MHz, DMSO-d6): δ = 11.62 (s, 1H, N-*H*), 11.48 (s, 1H, N-*H*), 8.23 (m, 2H, Ar-*H*), 8.04 (s, 1H, *C*-H) 7.89 (t, *J* = 8.9 Hz, 2H, Ar-*H*), 4.46 (m, 1H, O-*H*), 3.43 (m, 2H, -CH_2-), 2.67 (t, *J* = 7.5 Hz, 2H, -CH_2-), 2.27 (t, *J* = 7.5 Hz, 2H, -CH_2-), 1.71 (q, *J* = 6.6 Hz, 2H, -CH_2-). **^{13}C NMR** (Supplementary Figure 14) (100 MHz, DMSO-d6): δ = 175.2 (-*C*O-), 169.6 (*C*-NO_2), 148.0 (C_{Ar}), 141.3 (C_{Ar}), 140.4 (C_{Ar}), 128.2(C_{Ar}), 124.5(C_{Ar}), 60.7 (-CH_2-), 31.4(-*C*H_2-), 29.1(-*C*H_2-), 28.6(-*C*H_2-), 27.9(-*C*H_2-). **Mp**: 225-226 °C. **MS** (LC/MS, ESI neg.) m/z: 250 [$(M-H)^-$], (LC/MS, ESI pos.) m/z: 274 [$(M+Na)^+$], (expected m/z = 251.1).

Synthesis of PEG-aldehyde copolymer (6)

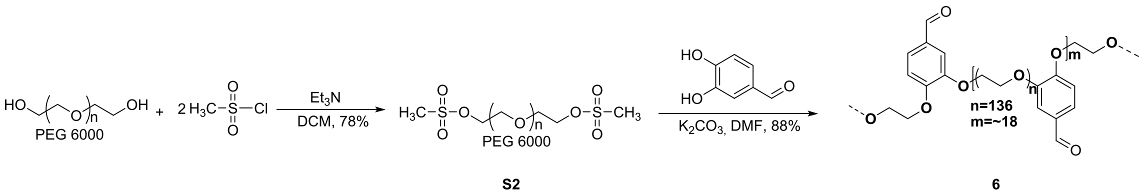

After functionalizing PEG-6000 with mesylchloride, the substitution reaction with 3,4-hydroxybenzaldehyde yielded the polydisperse PEG-aldehyde copolymer **6**.

Synthesis PEG-mesylate (S2)

PEG-6000 (molecular weight 5400 – 6600 g/mol) (50 g, 8.33 mmol) was dissolved in dry dichloromethane (300 mL), dry triethylamine (11.6 mL, 83.3 mmol) was added and the mixture was cooled on ice and brought under an argon atmosphere. Methanesulfonyl chloride (6.5 mL, 83.3 mmol) was added dropwise and the reaction mixture was stirred for 5 days at room temperature. Deionized water (200 mL) was added and the reaction was stirred vigorously. The water layer was extracted with dichloromethane (4 x 150 mL). The combined organic layers were dried over magnesium sulfate, filtered and most of the solvent was removed under reduced pressure. The concentrated reaction mixture was precipitated out in

diethyl ether (1.5 L) using a dropping funnel. The obtained powder was again dissolved in dichloromethane and precipitated out in diethyl ether (1.5 L) to obtain the PEG mesylate **S2** as a white powder (39 g, 6.3 mmol, 78% yield, 86% modification). The modification rate was determined by comparing the NMR peak of the mesyl group on the PEG-mesylate **S2** with the bulk peak of the PEG. PEG-6000 has on average 136 PEG-units per molecule; each PEG-unit has 4 protons, so the integral of the bulk PEG should be 545. The resulting integral of the mesylate peak is 5.13 whereas for 100% modification, the mesylate groups should give an integral of 6, so the modification rate is 5.13/6 is 86%. **^{1}H NMR** (Supplementary Figure 15) (400 MHz, CD_3OD): δ = 4.30 – 3.36 (m, 543H, bulk PEG-*H*), 3.00 (s, 6H, -CH_3).

Synthesis of PEG-aldehyde copolymer (6)

The PEG-mesylate **S2** (25 g, 4.1 mmol) was dissolved in dry DMF (250 mL) and the solution was bubbled through with argon. Oven dried K_2CO_3 (4.5 g, 32.5 mmol) and 3,4-dihydroxybenzaldehyde (0.843 g, 6.09 mmol) were added. The reaction mixture was stirred at 130 °C for 5 days. The reaction mixture was cooled down to room temperature. Deionized water (100 mL) was added and the reaction mixture was stirred for 30 minutes. An aqueous HCl solution (100 mL, 1 M) was added and the reaction mixture was extracted with dichloromethane (4 x 100 mL) and washed with deionized water (150 mL). The mixture was dried over Na_2SO_4 and filtered. Most of the solvent was removed under reduced pressure and the concentrated product was precipitated out in diethyl ether (1.5 L). The obtained product was dissolved in dichloromethane (150 mL) and precipitated out again in diethyl ether (1.5 L) to yield a light brown fluffy powder (22 g, 88%). **^{1}H NMR** (Supplementary Figure 16) (400 MHz, CD_3OD): δ = 9.82 (s, 3H, C*H*O), 7.45 (s, 6H, Ar-*H*), 7.01 (d, J = 8.0 Hz, 3H, Ar-*H*), 4.25 – 3.44 (m, 2148H, bulk PEG-*H*). GPC: 10 µL of 10 mg/mL polymers solutions were injected and eluted with milliQ water at 1 mL/min and 40 °C for 30 minutes (Supplementary Figure 17). Weighted average $M_n = 97 \cdot 10^3$ g/mol, weighted average $M_w = 109 \cdot 10^3$ g/mol, weighted average $M_z = 121 \cdot 10^3$ g/mol, PDI over all peaks = 9. These observations were confirmed in a DOSY experiment where we found a diffusion constant of $0.07 \cdot 10^{-10}$ $m^2 \cdot s^{-1}$, which corresponds to polyethylene glycol with a molecular weight of 100 $\cdot 10^3$ g/mol.

Synthesis of *cis*,*cis*-cyclohexane-1,3,5-tricarbohydrazide (7)

For a detailed procedure we refer to a protocol reported earlier in our group.[5] Trimesic acid (50.0 g, 238 mmol) and 10% palladium on activated charcoal (1.27 g, 11.9 mmol) were suspended in deionized water (300 mL) in a mechanically stirred Parr autoclave with a 1-liter volume. The autoclave was flushed with nitrogen gas. Hydrogen gas was added to the autoclave. The pressure was increased to 50 bar and the temperature to 150 °C. After

complete conversion was confirmed by NMR, the reaction mixture was filtered through a sintered funnel, concentrated under reduced pressure and dried in a vacuum oven to obtain a mixture of *cis*,*cis*- and *cis*,*trans*-isomers of 1,3,5-cyclohexanetricarboxylic acid. The obtained acid (37.2 g, 172 mmol) was dissolved in methanol (1 L). *p*-Toluenesulfonic acid monohydrate (1.48 g, 8.61 mmol, 5 ml%) was added to the solution and the reaction was stirred at 80 °C for 36 h. After NMR confirmed complete conversion of the starting compound, trimethylamine (10 mL) was added and the reaction mixture was concentrated under reduced pressure. Diethyl ether (1 L) was added and the reaction mixture was washed with water (1 L) and brine (1 L), dried with magnesium sulfate, filtered and concentrated under reduced pressure. Crystallization of the crude product in petroleum ether yielded the *cis*,*cis*-trimethylcyclohexane-1,3,5-tricarboxylate as colourless crystals. This carboxylate (40.2 g, 156 mmol) was dissolved in methanol (600 mL), hydrazine hydrate (141 mL, 64% in water) was added and the reaction mixture was stirred for 3 h at room temperature. The reaction mixture was concentrated under reduced pressure to obtain the *cis*,*cis*-cyclohexane-1,3,5-tricarbohydrazide **7** as a white powder (39.7 g, 154 mmol, 70% yield in two steps). **^{1}H NMR** (400 MHz, DMSO-d6): δ = 8.98 (s, 3H), 4.17 (bs, 6H), 2.11 (t, *J* = 11.8, 3H), 1.57 (d, *J* = 11.7, 3H), 1.45 (q, *J* = 12.6, 3H).

Synthesis of 3,4-bis(2-(2-methoxyethoxy)ethoxy)benzaldehyde 8

For a detailed procedure we refer to a protocol reported earlier in our group.[5] Diethylene glycol monomethyl ether (100 g, 832 mmol) was dissolved in tetrahydrofuran (THF) (500 mL) and triethylamine (168 g, 1.66 mol, 231 mL) was added. The reaction mixture was cooled to 0 °C and a solution of *p*-toluenesulfonyl chloride (132 g, 694 mmol) in THF (200 mL) was added dropwise (60 min). The reaction mixture was stirred overnight at room temperature after which most of the solvent was removed under reduced pressure. The crude product was dissolved in dichloromethane (DCM) (800 mL), washed with deionized water (1 L) and extracted with DCM (3 x 500 mL). The combined organic layers were washed with brine (200 mL), dried over magnesium sulfate, filtered and concentrated under reduced pressure to obtain the tosylate product as a pale yellow oil that crystallized out as a light yellow solid (181 g, 660 mmol, 95%). The 2-(2-methoxyethoxy)ethyl tosylate (40.0 g, 146 mmol) was dissolved in dry DMF (200 mL), oven dried potassium carbonate (40.3 g, 292 mmol) was added and the reaction was brought under an argon atmosphere. 3,4-Hydroxybenzaldehyde (9.16 g, 66.3 mmol) was added and the reaction was stirred overnight at 90 °C under an argon atmosphere. The reaction mixture was cooled to room temperature, 1 M HCl (200 mL) was added and the mixture was extracted with ethyl acetate (4 x 250 mL). The combined organic layers were washed with a saturated sodium bicarbonate solution (5 x 200 mL) and brine (5 x 200 mL), dried over magnesium sulfate, filtered and concentrated under reduced pressure to

yield the product as a yellow oil (21.3 g, 62.1 mmol, 94%). **^{1}H NMR** (400 MHz, $CDCl_3$): δ = 9.82 (s, 1H), 7.42 (m, 2H), 6.98 (d, *J* = 11.6, 1H), 4.19–4.27 (m, 4H), 3.89 (t, *J* = 6.8, 2H), 3.88 (t, *J* = 6.4, 2H), 3.70–3.75 (m, 4H), 3.53–3.58 (m, 4H), 3.37 (s, 6H).

Determination pseudo-first-order rates hydrazone formation 5

Pseudo-first-order conditions were obtained by using a 5 times excess of aldehyde **4** (0.5 mM) with respect to hydrazide **3** (0.1 mM) during the reactions.

The integrated rate law for first-order reactions is:

$$[P] = [\mathrm{P}]_0 \cdot e^{k_1 \cdot t} \qquad \text{Equation (1)}$$

$$\ln[P] = \ln[P]_0 + k_1 \cdot t \qquad \text{Equation (2)}$$

$$[P] = \frac{A}{\varepsilon_{350} \cdot l} \qquad \text{Equation (3)}$$

In which $[P]$ is the concentration of hydrazone **5**, determined by dividing the absorbance measured at 350 nm during the reaction with the extinction coefficient of hydrazone **5** at 350 nm. k_1 is the first-order rate constant (s^{-1}). A is the absorbance at 350 nm measured during the reaction. ε_{350} is the extinction coefficient of hydrazone product **5** at 350 nm. Plotting ln[P] versus the time (s) gives the first order rate constant. The graphs were fitted using linear regression in Origin Pro 2015. For the uncatalyzed reaction and the reaction with pro-aniline **1**, the first 10 h of the reaction were taken to determine the reaction rate constants. For the reaction in the presence of activated pro-aniline **1** and for the reaction catalysed by aniline **2**, the first 15 minutes were used to determine the reaction rate constant (Supplementary Fig. 8).

UV/vis hydrazone formation blank experiments

After activation of pro-aniline **1**, the reaction rate of hydrazone formation was lower than when using native aniline **2**. In an attempt to explain this loss of catalytic activity we investigated the influence of H_2O_2 and of boric acid to aniline **2**, but could not find any loss in catalytic activity (Supplementary Fig. 3c, d). Reaction conditions: 0.1 mM hydrazide **3**, 0.5 mM aldehyde **4**, 0.5 mM boric acid, 2.5 mM H_2O_2, 0.5 mM aniline **2** in 20% DMF in phosphate buffer (100 mM, pH 7.4). Even though we confirmed complete conversion of pro-aniline **1** after addition of more than 1 equivalent of H_2O_2 and we detected aniline **2** after an overnight hydrazone reaction in the presence of pro-aniline **1** and H_2O_2 (Supplementary Fig. 4), it might be the case that some aniline **2** is lost or degraded in the process. Furthermore, the pH was monitored for the reaction with pro-aniline **1** (0.5 mM) and H_2O_2 (2.5 mM), every 10 minutes for the first 2 h and once after 18 h. No pH change was observed during the reaction: the pH remained stable at a pH of 8.0. The solvent system itself, 20% DMF in

phosphate buffer (100 mM, pH 7.4) gives a pH of 8.0, which indicates that the phosphate buffer concentration is sufficient to buffer the reaction mixtures.

Supplementary References

1. Nuñez, S. A., Yeung, K., Fox, N. S., Phillips, S. T. A structurally simple self-immolative reagent that provides three distinct, simultaneous responses per detection event. *J. Org. Chem.* **76,** 10099-10113 (2011).

2. Wipf, P., Maciejewski, J. P. Titanocene(III)-catalyzed formation of indolines and azaindolines. *Org. Lett.* **10,** 4383-4386 (2008).

3. Chung, C., Srikun, D., Lim, C. S., Chang, C. J., Cho, B. R. A two-photon fluorescent probe for ratiometric imaging of hydrogen peroxide in live tissue. *Chem. Commun.* **47,** 9618-9620 (2011).

4. Popiołek, Ł., Biernasiuk, A., Malm, A. Synthesis and antimicrobial activity of new 1,3-thiazolidin-4-one derivatives obtained from carboxylic acid hydrazides, *Phosphorus, Sulfur, Silicon Relat. Elem.* **190,** 251-260 (2015).

5. Poolman, J. M. *et al.* Variable gelation time and stiffness of low-molecular-weight hydrogels through catalytic control over self-assembly. *Nat. Protoc.* **9,** 977-988 (2014).
